# Mental Imagery and Brain Regulation—New Links Between Psychotherapy and Neuroscience

**DOI:** 10.3389/fpsyt.2019.00779

**Published:** 2019-10-30

**Authors:** Leon Skottnik, David E. J. Linden

**Affiliations:** School for Mental Health and Neuroscience, Faculty of Health, Medicine and Life Sciences, Maastricht University, Maastricht, Netherlands

**Keywords:** mental imagery, emotion-regulation, psychotherapy, neuromodulation, neurofeedback, real-time fMRI, brain stimulation

## Abstract

Mental imagery is a promising tool and mechanism of psychological interventions, particularly for mood and anxiety disorders. In parallel developments, neuromodulation techniques have shown promise as add-on therapies in psychiatry, particularly non-invasive brain stimulation for depression. However, these techniques have not yet been combined in a systematic manner. One novel technology that may be able to achieve this is neurofeedback, which entails the self-regulation of activation in specific brain areas or networks (or the self-modulation of distributed activation patterns) by the patients themselves, through real-time feedback of brain activation (for example, from functional magnetic resonance imaging). One of the key mechanisms by which patients learn such self-regulation is mental imagery. Here, we will first review the main mental imagery approaches in psychotherapy and the implicated brain networks. We will then discuss how these networks can be targeted with neuromodulation (neurofeedback or non-invasive or invasive brain stimulation). We will review the clinical evidence for neurofeedback and discuss possible ways of enhancing it through systematic combination with psychological interventions, with a focus on depression, anxiety disorders, and addiction. The overarching aim of this perspective paper will be to open a debate on new ways of developing neuropsychotherapies.

## Introduction

The neural mechanisms of psychotherapy have been investigated with methods of functional imaging for over 20 years (1–4). This line of investigation has fascinated psychotherapists and neuroscientists alike and has the potential of aiding the design of new therapies that are guided by neural mechanisms, as well as aiding the allocation of patients to particular treatment modalities. It is also a particularly challenging area of research because most therapies target a range of interacting processes, resulting in complex changes in neural activation, which are also difficult to study over the generally long timeframes of psychological treatment programmes. One success story in this respect has been the elucidation of mechanisms of mental imagery—in general and in its specific relevance for emotional states. The opportunity to study the neural substrates of mental imagery, which came with the development of non-invasive functional imaging methods, particularly functional magnetic resonance imaging (fMRI), has been parallel by an increasing interest in its use in modern cognitive and behavioural therapy approaches. An overview of this field is thus topic for a volume on mechanism of psychotherapy. In addition, the authors, and a growing international community of clinicians and researchers, have developed an interest in the combination of mental imagery with neuromodulation techniques, particularly neurofeedback. We will argue that the advances in the neuroscience of mental imagery and the increasing evidence for its utility particularly in affective and anxiety disorders now create a unique opportunity to exploit the enhancement of imagery approaches through neuromodulation (and vice versa) for the development of new integrated treatment tools, which may initially focus on depression, post-traumatic stress disorder, and specific phobias, but can, in principle, also be developed for a much wider range of psychological disorders.

## Mental Imagery-Based Treatments and Implicated Brain Networks

While psychodynamic treatment approaches have analysed the symbolic properties of mental images for decades, cognitive therapies have recently developed efforts to incorporate mental imagery in order to alter affective states and modify their cognitive context (see the study by Edwards (5), and note, for example, the study by Clyne (6) for an active psychodynamic imagery approach). Independently, different mental imagery interventions were developed that aimed to facilitate the effects of mental imagery trough neurofeedback (7–11). By summarising the neuroscientific evidence on mental imagery with and without neurofeedback, this review highlights the potential for cross-disciplinary treatments that combine psychological and neuroscientific tools strategically.

Firstly, the main domains of mental imagery in psychotherapy will be introduced and neuroscientific evidence on their working mechanisms will be discussed. Secondly, psychiatric applications of neurofeedback and the main working mechanisms of neurofeedback trainings will be reviewed. Thirdly, existing evidence on interactions between psychotherapy and neurofeedback approaches will be summarised and future pathways of combining psychotherapy with neurofeedback will be discussed, as well as the potential benefit of brain stimulation techniques.

### Mental Imagery in Psychotherapy

#### Endogenous Generation of Emotional States

A crucial feature of mental images is their ability to induce emotional states (12–14). Taking into account that especially mood and anxiety disorders are marked by dysfunctions of the emotional system, this property of mental imagery has been repeatedly used to induce changes in the affective symptoms of these disorders.

A main category of mental imagery applications used to achieve this consists of repeated mental imagery with positive valence. Repeated positive imagery has been shown to increase the tendency to interpret ambiguous situations as more positive and induce positive mood in healthy participants (15, 16). As depression in particular is marked by pronounced negativity biases (see the review by Gotlib and Joormann (17) for an overview), i.e., the tendency to interpret ambiguous situations as more negative, it can be expected that positive mental imagery will improve depressive symptoms through strengthening positive affective reactions. Accordingly, a recent clinical trial has evaluated positive mental imagery as being effective in reducing such negative biases in depression (18).

Another main category of mental imagery applications consists of mental imagery of specific, fear inducing mental objects or contexts, i.e., imaginal exposure. For example, through imagining fear inducing stimuli that trigger phobic reactions repeatedly, patients can desensitize. This ‘imaginal exposure’ approach has been successfully implemented in the treatment of a variety of phobias (19–22). Symptoms of other anxiety disorders have also been shown to be significantly attenuated by imaginal exposure, including obsessive-compulsive disorder (23–25) and post-traumatic stress disorder (26, 27).

#### Controlling Mental Images and Their Cognitive Context

In addition to the two main valence categories of mental imagery (enhancing positive emotions, or imaginal exposure to negative material), some treatment approaches entail switches between negative and positive imagery, for example, imagery rescripting (28–32) or guided imagery (33–35). Switching valence is thereby usually achieved by either changing a mental image to an image with more positive features or wholly replacing it with another image (36). As negative mental imagery constitutes a crucial feature of major psychopathologies (36), it seems not surprising that such substitutions or alterations of negative mental images can be effective across a range of disorders (see the studies by Holmes et al. (37) and Stopa (38) for an overview), including post-traumatic stress disorder (PTSD) (30, 32, 39) and depression (40, 41).

While rescripting aims to alter the content of mental images and, accordingly, also their emotional effect (37, 42), strategies that focus on cognitive control instead aim at changing the interpretations of (mental) images, i.e., their cognitive context (42–44). Here, notably cognitive reappraisal has been widely used to alter the cognitive context of emotion-inducing material (45, 46). Reappraisal has thereby repeatedly been shown to function as an effective emotion regulation strategy, compared to other top-down emotion-regulation strategies as, for example, active suppression (47–49).

### Brain Networks Recruited During Mental Imagery

**Figure 1:**
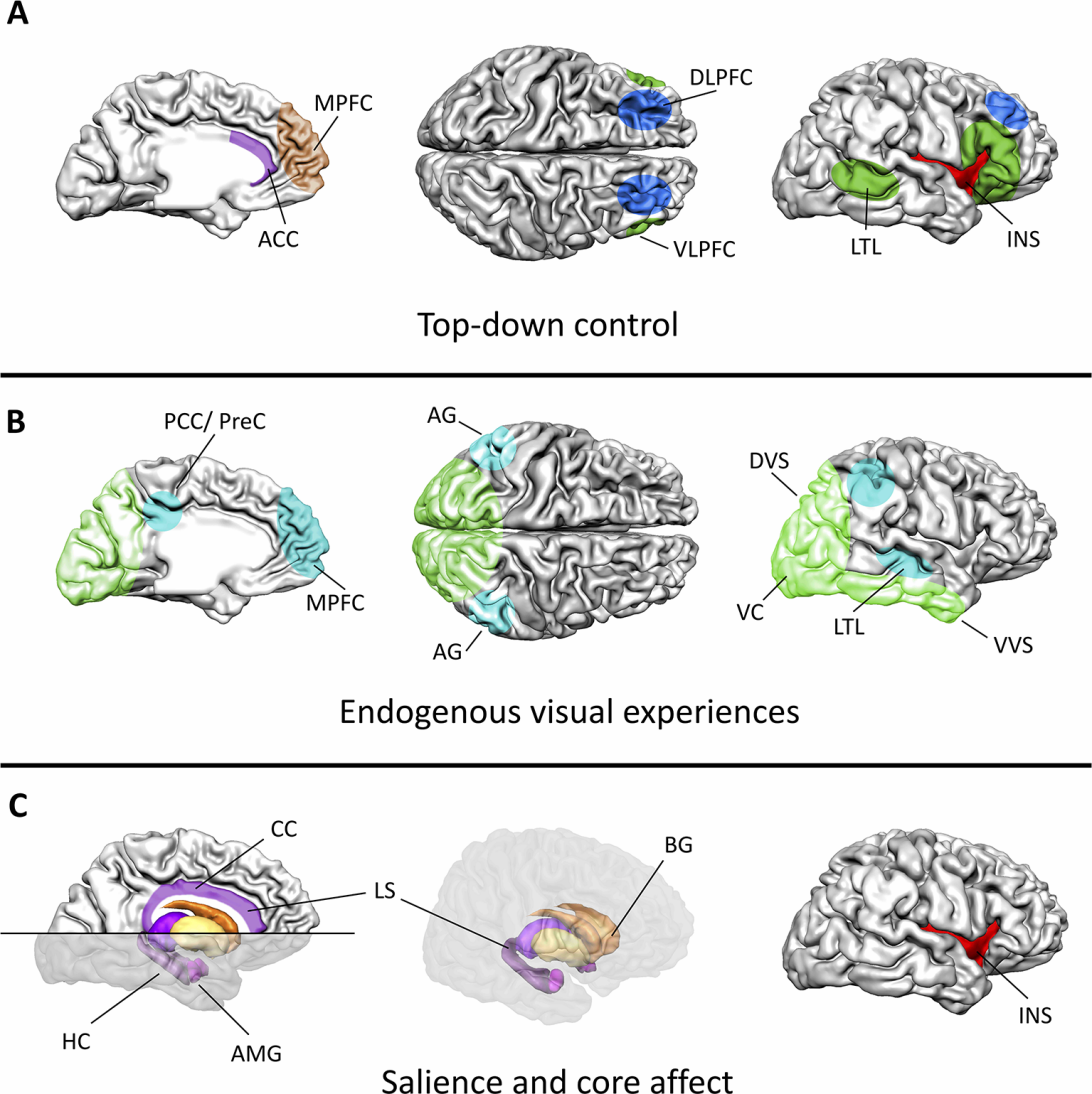
Major functional components and associated brain systems of mental imagery during emotion regulation. **(A)** Cognitive control during mental imagery treatments relies on a distributed cortical network. The dorsolateral PFC and lateral parietal cortex (blue) control mental resources, while the semantic interpretation of the internal state is associated with activation in the ventrolateral PFC and the lateral temporal cortex (dark green). The anterior cingulate cortex (ACC, purple) and the medial PFC (brown) monitor the ongoing processes. The ACC together with the insula (red) thereby function as major connecting hubs between bottom-up saliency information and top-down attentional control. **(B)** Visual experiences during mental imagery mimic actual perception of external stimuli in the visual system (light green). The sensory content of a mental image is thereby activated across different levels of the visual processing hierarchy, ranging from low level visual areas to associate cortices. Modulations in the default-mode-network (turquoise) indicate increased processing of the endogenously generated information. **(C)** Particularly, the limbic system (purple), the basal ganglia (orange/yellow), and the insula (red) encode the hedonic value, arousal, and salience of the visual experience. This information can directly affect ongoing learning processes as particularly the hippocampus is crucially involved in encoding and retrieving emotional memories. The insula, the anterior cingulate cortex, the amygdala, and the ventral basal ganglia form a network that encodes the salience of a generated experience. As a connecting hub between various systems the insula constitutes a key region for introspection during mental imagery. ACC, anterior cingulate cortex; AG, angular gyrus; AMG, amygdala; BG, basal ganglia; DLPFC, dorsolateral PFC; DVS, dorsal visual stream; HC, hippocampus; INS, insula; LS, limbic system; LTL, lateral temporal lobe; MPFC, medial PFC; PCC/PreC, posterior cingulate cortex/precuneus; VC, visual cortex; VLPFC, ventrolateral PFC; VVS, ventral visual stream; CC, cingulate cortex.

Considering that the main focus of mental imagery applications in psychotherapy lies in influencing emotional processes, an understanding of the neural basis of emotions is fundamental for understanding the neural effects of mental imagery. The current section will therefore provide an overview on the brain systems connecting mental imagery to affective processes (**Figure 1**).

#### Emotional Experiences Are Associated With Widely Distributed Brain Activation

With the immense growth of available neuroimaging data during the last century, a vast body of empirical evidence has grown that helps to relate activation in defined brain structures to emotional experiences. Studies that focused on specific involvement of certain brain regions showed some correspondence between brain structures and emotional states. The amygdala, for example, appears to reliably activate during the experience of fear (50–52). Such findings are in accordance with a body of neuropsychiatric evidence suggesting that lesions in these areas lead to attenuated fear responses (53, 54). Similar findings have been obtained for many other emotional states, e.g., for the insula as being crucial in experience for disgust (55–57) or the orbitofrontal cortex for anger (58, 59).

Although such findings show that activation in certain brain areas can reflect subjective psychological experiences of emotions, they do not imply that activation in these brain structures is necessarily specific to a particular emotional state. Meta-analysis combining data of different emotional states could reliably show that the amygdala contributes to various different emotions (60, 61), with negative as well as positive valence. Such findings support contemporary constructivist approaches, which constitute that emotional experiences do not emerge from increased activation of single brain structures, but rather from pattern of activation distributed across brain regions that subserve more general functions (see, for example, the studies by Barrett (62) and Lindquist and Barrett (63)). In constructivist approaches, a brain area contributes to an emotional experience by giving rise to one of the many subprocesses from which a holistic emotional experience is constructed. The amygdala, for example, has been implicated in indicating the salience of an emotional stimulus (64, 65) and its involvement in fear has thereby been related to the high salience of fear inducing stimuli. Accordingly, more general functions for various other structures involved in core affect have been suggested (see the study by Lindquist and Barrett (63) for an overview).

Notably, emotional experiences are additionally accompanied by activation in areas that are not directly implicated in core affect (60, 61, 66–68). Across emotional categories, activation increases have been observed in prefrontal areas related to higher order cognitive processes (see the studies by Storbeck and Clore (69) and Duncan and Barrett (70)), the memory system (notably, the medial temporal lobe complex, see the studies by Squire and Zola-Morgan (71); Dolcos et al. (72); Squire et al. (73), and Eichenbaum et al. (74)) or sensory processing (see, for example, the study by Saarimäki et al. (75)), and particularly modulations in visual cortex activity have been observed reliably (61, 76–78). It has therefore been repeatedly argued that emotional experiences arise from distributed neural representations that include affective, cognitive as well as sensory components (61, 69). Particularly, this embeddedness of the affective system into cognitive and sensory networks provides the possibility for mental imagery approaches to alter emotional states by targeting the sensory or cognitive components of emotions.

#### Brain Activation During Mental Imagery—Similarities With Visual Perception

In accordance with the notion that emotional states are linked to associated sensory experiences, it has been suggested that, during mental imagery, especially, the sensory aspects of a mental image (in contrast to the semantic information contained in it) are fundamental in evoking emotions (36). Indeed, when comparing emotion induction through language to emotion induction by mental imagery, mental imagery evokes stronger emotional responses even when the semantic content is matched between modalities (37, 79). In contrast to actual visual perception, mental imagery is not triggered by external visual stimuli and constitutes a purely mental operation. Interestingly, patterns of brain activation in the visual system during mental imagery resemble activation of weak external visual stimuli (see the study by Pearson et al. (80) for an overview).

A considerable body of experimental psychology and cognitive neuroscience studies suggests that mental imagery can be considered as a form of top-down induced (i.e., internally generated) visual perception, although the exact neural mechanisms are the matter of an ongoing debate. Early fMRI studies revealed reliable brain activation during mental imagery in higher level visual areas, i.e., areas in which visual information is combined to comprehensive visual features (notably the associate visual cortex, early studies by D’Esposito et al. (81); Goebel et al. (82), and Knauff et al. (83). Activation in V1 in turn, i.e., the earliest cortical processing stage of visual information, has also been observed during mental imagery (84–86) but appears to be less pronounced (86) and the ability to form vivid mental images remains preserved after extensive lesioning of V1 (87, 88), supporting that V1 incorporates a less crucial function for the formation of mental images than higher level areas of the visual stream.

Notably, while emotion-related brain activation has been observed in the whole visual cortex, findings for V1 are strongly modulated by the use of visual stimulation in experiments (60). In contrast, higher level visual areas reliably activate also when no visual stimulation is provided (61), suggesting that they serve a more general function in relation to emotions. Crucially, it has been shown that mental imagery is comparably effective as other common mood induction procedures in evoking emotional states (12, 13, 89), underlining that mental imagery can modulate the affective system effectively.

In addition to the cortical pathway which processes complex visual information and gives rise to conscious visual percepts, a subcortical route of visual processing exists for simple, evolutionally meaningful stimuli such as snakes or spiders (90–93). Although the influence of mental imagery on such reflexive sensory-affect connections has not yet been investigated thoroughly, the effectiveness of mental imagery as treatment of the phobias associated with these groups of animals (see, for example, the studies by Lang et al. (94) and Hunt and Fenton (95)) suggests that, at least, the emotional reactivity of subcortical areas involved (prominently the amygdala) can be modulated through mental imagery. However, further research is needed to determine the influence of mental imagery on such basic visual triggers of the affective system. Neurofeedback and other neuromodulation strategies may play a particular role for the synergistic targeting of such limbic circuits.

#### Memory and Cognitive Control Systems Determine the Context of a Mental Image

While the affective and sensory experiences are intertwined, their relationship is modulated by past experiences and ongoing cognitive processes (96). Taking into account that retrieval of autobiographical memories is commonly associated with mental imagery, an overlapping neural mechanism of episodic memory and mental imagery has been suggested (79, 97). In parallel, it has been shown that mental simulations of fictional events share an extensive neural basis with episodic memory retrieval, including the default-mode and frontoparietal control network (FPCN) (see the studies by Botzung et al. (98); Gerlach et al. (99), and Benoit and Schacter (100)).

In clinical conditions, this interconnectedness of episodic memory and mental imagery appears to be of particular relevance. Several common psychopathologies are characterised by intrusive mental images of emotionally loaded past experiences, and in the case of PTSD, they constitute a core symptom (see the study by Hackmann and Holmes (101)). Brain activation in relation to such intrusive mental images in PTSD (“flashbacks”) has been associated with increased activation in higher-order visual areas in comparison to ordinary episodic memoires (102), supporting the clinical relevance of connections between mental images and the memory system.

While particularly autobiographical experiences have been suggested to contribute to the effects of mental imagery in emotion regulation (79), networks involved in processing the current cognitive context of a percept also strongly contribute to its effect on the affective system (103–105). In this context, anterior prefrontal areas along the midline (particularly the ventromedial PFC and anterior cingulate cortex) have been suggested to integrate incoming information from the limbic system and sensory areas with higher level cognitive goals (96). Lateral prefrontal and parietal areas, in turn, have been suggested to subserve primarily executive functions in the context of emotion regulation. Particularly, activation in the FPCN has been shown to relate to success in emotion generation (106). Interestingly, the FPCN has also been shown to be selectively involved in retrieving visuospatial features of episodic memory (107), supporting the interconnectedness of episodic memory, mental imagery, and emotion.

#### Different Psychotherapeutic Approaches Target Different Neural Subsystems

Overall, these findings suggest that the neural basis of mental imagery, especially in the context of emotion regulation, spans several different functional domains. Although the recruited networks are highly interconnected and partly overlapping, the different mental imagery-based treatment approaches put more weight on certain subsystems.

Approaches that focus on endogenous generation of emotions through mental imagery (e.g., by using mental imagery to induce positive mood or negative mental images for desensitization) firstly aim at modulating the affective core system. Such endogenous generation of emotions has been shown to involve distributed neural components that interact to create an emotional experience and are shared between positive and negative valance, as well as levels of arousal (106).

For generating the affective core state, particularly the salience network (i.e., a network crucial in determining the saliency of external or internal events) together with the limbic system appears to be crucial. The actual representation of an emotional experience, i.e., an affective state coupled to its mental content, is associated with coactivation of limbic areas, particularly the amygdala and the striatum, and areas related to inward directed attention, particularly the default mode network. Such representations of an emotional state additionally show modulations specific to the mental modality used for emotion generation, i.e., they are accompanied by different activation for mental imagery of visual/autobiographical memories, bodily experience, and semantic strategies. Additionally, before the representation of an affective experience is created, the FPCN has been suggested to initiate the emotion generation processes and, afterwards, contribute to the maintenance of the generated mental representation (106). Due to the role of the FPCN in executive functions and attention, it likely mobilizes and bundles the mental resources necessary to engage in the emotion generation procedure.

While endogenous emotion generation thereby also involves top-down control systems, other treatment approaches target higher order cognitive processes more explicitly. Notably, for cognitive reappraisal, several studies have tried to disentangle the involved brain systems (early studies by Beauregard et al. (108) and Ochsner et al. (109); see also the study by Buhle et al. (110) for an overview). Cognitive reappraisal implies that an emotionally salient event already is present (as the object of the reappraisal, which is represented physically or recalled in memory), this approach does not focus on initiating activation in the core affective system, but rather on altering activation in areas contributing to the cognitive context of the emotional event (which then leads to modulations in the affective core system). In this process, the ventrolateral PFC and the lateral temporal cortex have been suggested to contribute to selecting and representing the semantic context, while the dorsolateral PFC and lateral parietal cortex support shifting the semantic context by modulating the attentional focus towards particular aspects of the mental event and controlling working memory content. In addition, the ACC and the medial PFC have been suggested to monitor ongoing affective and cognitive processing and integrate information from both systems to execute appropriate top-down control (43, 96, 110–112). Most neuroimaging work converges to suggest that cognitive reappraisal is associated with downregulation of the amygdala (110), thereby potentially decreasing the salience of the emotional event.

### Neurofeedback Interventions

As mental imagery and emotion regulation recruit defined brain systems, techniques that modulate brain activation can be implemented to support the neural processes that take place in these networks. Additionally, especially neuroimaging-based techniques can thereby provide valuable information on the neural effects of the mental operations that are performed. Neurofeedback approaches combine these two aspects. During a neurofeedback intervention, neuroimaging is applied in order to measure relevant markers of brain activation and feed this information back to participants. By providing neurofeedback during mental imagery, participants thereby become enabled to adapt their mental imagery strategies based on whether the strategies successfully contribute to achieving a desired brain target state.

For several decades, neurofeedback has been created from electrophysiological brain signals acquired through electroencephalography (EEG) (see the study by Kamiya (113) for an overview). While EEG can provide temporally accurate feedback and is accessible to communities, it is a matter for debate whether it can provide reliable signals from deeper brain structures (Nunez et al. (114); Babiloni et al. (115); Burle et al. (116), but see the study by Keynan et al. (117)). Functional magnetic resonance imaging (fMRI) on the other hand, can more accurately narrow down the location in the brain from which neurofeedback is provided and can create neurofeedback from subcortical areas (see the study by Auer and Auer (118)).

#### Clinical Applications of Neurofeedback

Neurofeedback can be incorporated in psychotherapy because of its active use of psychological techniques, which can range from classical and operant conditioning to explicit imagery and cognitive strategies. This section will review the development of clinical neurofeedback based on fMRI for psychiatric applications. It will focus on fMRI-based neurofeedback rather than incorporate the much larger field of EEG-based neurofeedback because the design of fMRI studies, targeting specific brain regions or networks, has generally followed the considerations of pathophysiological or compensatory networks outlined in the previous sections of this review and has often explicitly incorporated elements of mental imagery.

The clinical development of fMRI-neurofeedback (fMRI-NF) started in 2005 with the publication of a study in patients with fibromyalgia (119). Although conceptually, there should be great promise of such a self-regulation training in patients with chronic pain syndromes relatively little work has followed up on this. In 2011, we published our first study on fMRI-NF in neurorehabilitation, targeting the supplementary motor area (SMA) in Parkinson’s disease (120), which was followed up by further studies targeting SMA in neurodegenerative diseases (121, 122). In terms of psychiatric applications, patient studies have mainly been conducted in depression, anxiety disorders, and addiction. In a pilot study of fMRI-NF in depression, we trained patients with depression to upregulate brain networks responsive to positive affective stimuli. This paradigm was modelled on our previous work with healthy participants, which had shown that the neurofeedback component (compared to imagery alone) is required for reliable control over emotion networks (123). The eight patients who underwent this fMRI-NF protocol for four sessions improved significantly on the 17-item Hamilton Rating Scale for Depression, and this clinical improvement was not observed in eight control patients who engaged in a protocol of positive emotional imagery (matched to the fMRI-NF protocol for intervention and assessment times and affective stimuli) outside the scanner (124). In a follow-up randomised controlled trial (RCT) with a similar protocol, we found similar levels of clinical improvement; however, this was also seen for a control intervention using neurofeedback of the parahippocampal place area, an area involved in the processing of scenes and places (125).

A recent RCT pitting upregulation training of the amygdala against upregulation of a control area (the intraparietal sulcus region) found clinical improvement in patients with depression that was significantly stronger in the active (amygdala upregulation) than the control intervention (126). Thus, there is now evidence from RCTs to suggest that fMRI-NF targeting brain networks of emotions may have benefits for patients with depression although it is less clear how specific the protocols/trainings have to be to be effective.

In the area of anxiety disorders, several studies have investigated self-regulation training of the amygdala for PTSD (127–131), and another line of research is targeting the anterior cingulate cortex (131). These have mostly been feasibility studies and information about clinical outcomes is not yet available. Interestingly, both amygdala upregulation (during positive autobiographical memory) (129) and downregulation (during confrontation with traumatic experience) (130) have been employed. Pilot studies have also been conducted in specific phobia (132) and obsessive compulsive disorder (OCD) (133). FMRI-NF has also been piloted for several substance use disorders (134–137) and formal clinical trials are under way(138, 139).

#### Brain Mechanisms Targeted by Neurofeedback Trainings

Mental imagery is one of the most common strategies reported by participants training to control the neurofeedback signal and often explicitly suggested as a potential strategy by the investigators. For this reason, the same general networks related to self-regulation, emotion, and visual imagery are modulated during these neurofeedback interventions as discussed for the psychotherapeutic applications of mental imagery (124, 140, 141). As a crucial difference to conventional mental imagery approaches, neurofeedback training additionally entails providing information on a subcomponent of these networks to the participant.

Across different psychiatric populations, a common target of fMRI neurofeedback trainings is core emotional areas (**Figure 1**). By providing participants with the opportunity to identify mental strategies that boost/decrease activation in these regions, participants can learn to gain control over their emotional system. This is usually achieved by training participants to increase brain activation in relevant areas during positive mental imagery (123–125, 126, 142) or to decrease activation in areas responsive to negative affective stimuli (143, 144). While neurofeedback provides information on whether a mental strategy is effective in modulating the neural basis of an emotional state, it has also been shown that neurofeedback at the same time stimulates the reward system (141, 145, 146) thereby potentially reinforcing the desired neural target state additionally (see the study by Sitaram et al. (147) for an overview on prominent theoretical accounts on the working mechanisms of neurofeedback).

In addition to inducing changes in the affective core system, neurofeedback approaches can also facilitate specific mental content during mental imagery. In their recent neurofeedback trial in depression, Young et al. (126) showed that positive autobiographical imagery with neurofeedback from the amygdala selectively increases recall of positive memories, even when controlling for general effects of mental imagery. Other neurofeedback approaches have additionally shown that even specific properties of visual representations can be reinforced by neurofeedback. By providing feedback from pattern of activation (rather than only from decreases or increases in averaged regional brain activation), Shibata et al. (148) found evidence that neurofeedback can induce early visual cortex activation related to basic properties of visual stimuli, even in the absence of visual stimulation. While this study by Shibata et al. (148) demonstrated that neurofeedback can modulate low level features of internally generated visual representations, Taschereau-Dumouchel et al. (149) additionally demonstrated that neurofeedback training can also decrease emotional responses to visual-memory representations of fear inducing animals (see further discussion of this approach in the next section). Overall, the available evidence suggests that neurofeedback can indeed modulate specific mental representations, including perceptual, affective as well as memory components of emotional states.

Prefrontal and parietal control regions implicated in mental imagery are also recruited during most neurofeedback approaches (140, 145), which may reflect the use of mental imagery strategy in neurofeedback training (although other cognitive component processes may also recruit parts of this network). In comparison to mental imagery by itself, however, neurofeedback additionally modulates activation in areas related to reward learning, notably, the striatum (141). While neurofeedback can therefore also be expected to generally reinforce top-down control during mental imagery, it should specifically facilitate interactions between control systems and the neurofeedback target region. Studies investigating changes in brain connectivity have supported this notion by repeatedly showing training-related changes in connectivity between neurofeedback target regions and cortical control regions (150–152). Beyond the general effects of neurofeedback on brain connectivity, contemporary neurofeedback approaches can also target top-down control directly through neurofeedback, for example, by providing feedback from connectivity between control areas and affective core regions (153, 154).

### Systematic Combinations of Neurofeedback With Psychological Interventions

By its very nature, a neurofeedback training can be thought of as a psychological as much as a physiological intervention. Many studies use functional localiser procedures, which aim to capture the psychological processes involved in the disorder, such as anhedonia/reduced reward sensitivity in the case of depression, excessive cue reactivity/salience in the case of addictions or excessive fear responses in the case of anxiety disorders. Furthermore, many protocols involve instructions or explicit mood induction strategies, for example, to engage with positive autobiographical memories (155), or offer patients the opportunity to use cognitive strategies learnt in the course of other treatment programmes to try and aid the self-regulation of disease-relevant circuits. In these approaches, mental imagery is commonly an integral part of the intervention. Participants gain control over the neurofeedback signal by identifying the mental content which most strongly affects the neural processes that is regulated. Finally, some studies have used homework exercises to transfer the mental strategies used in the MRI environment into everyday settings, which may be a crucial step to ensure sustainability of any clinical effects.

There is also often a strong element of personalisation. The functional localisers commonly use material that is relevant to the disease process or the putative functional deficit such as positive affective pictures for localisation of emotionally responsive regions in depression (124), contamination scenes in contamination anxiety (133), pictures of spiders in arachnophobia (132), pictures of food to train regulation of food craving (156), or pictures of addictive cues for use in addiction (157). Although generic stimuli from general picture databases such as the International Affective Pictures System will often be sufficient to induce the desired activation patterns (and accompanying physiological responses, e.g., arousal) for many of these protocols, a personalised approach is preferable. For example, if the aim of the neurofeedback training is to target dysfunctional activation patterns that contribute to clinical symptoms directly, the protocol will generally involve induction of these symptoms during the scanning with personalised stimuli. A classical example is OCD, where patients might be asked to take pictures of the scenarios that trigger their symptoms, which can then be used for symptom induction in the MRI environment (158). This approach is parallel to the use of exposure to triggering events in psychological interventions for PTSD. The use of such (personalised) disease-relevant stimuli is not confined to the functional localiser procedure.

In some protocols, such stimuli (images, scripts) are also presented during the neurofeedback runs. One example is the instruction to downregulate amygdala activity during exposure to trauma words (128). In such a scenario, fMRI-NF is incorporated in a broader programme of personalised extinction therapy. Neurofeedback has also been used to pilot a virtual exposure training that would not involve actual presentation of symptom-provoking stimuli but only the reinforcement of the related brain activation patterns. This approach is based on a method called decoded neurofeedback, which entails identification of multivoxel patterns associated with particular stimuli or mental states, which are then used as neurofeedback target (10). In this decoded neurofeedback approach, participants are generally not explicitly told about specific mental strategies, and indeed the basic concept entails that they have no explicit knowledge of the nature of the brain activation patterns being reinforced. In the virtual exposure training, participants then train to increase the occurrence of brain patterns associated with aversive stimuli (which could have been localised in other participants, obviating the need for ever exposing the patient directly to the aversive stimulus) in an operant conditioning task. A pilot study with this paradigm in people showing high subclinical fear for specific animal categories has yielded promising results in terms of attenuation of arousal responses (149, 159).

Neurofeedback treatments, particularly those employing real-time fMRI feedback, thus intrinsically incorporate elements of psychological therapy (exposure, reappraisal and other cognitive strategies, mental imagery) to varying degrees. Indeed, a key element for any clinical implementation of such a technique would seem to be its integration with psychological therapy and training programmes, both in order to harness synergies and ensure sustainability of any effects.

## Challenges and Future Directions

### Neurofeedback and Mental Imagery

The research discussed in this review highlights the complexity of the neural systems recruited during mental imagery treatments, but also convergent pathways observed across studies that can be considered as well-validated targets for neurofeedback (**Figure 1**). This review has focused on the visual system because its involvement in mental imagery has been studied in greatest detail, and most clinical applications of mental imagery focus on the visual domain.

However, extensive evidence also suggests that other forms of imagery such as somatosensory or auditory imagery share a defined neural basis with their respective perceptual system (160–163). Additionally, it has been shown that these imagery modalities also modulate affective experiences and their neural basis (106, 164). Notably, individual differences exist with regard to which imagery modality participants prefer, and these differences are associated with differences in effectiveness of mental strategies (164). Future studies will have to evaluate in how far individual patients react better to certain types of mental imagery treatment. Here, neuroimaging and particularly neurofeedback could help to determine whether a certain type of mental imagery is more effective for modulating a neural target in an individual participant compared to other types of mental imagery. This appears to be especially relevant as a major limitation of mental imagery is that mental imagery success is experienced subjectively and mostly reported retrospectively. Such self-report procedures are generally affected by biases, especially when performed retrospectively (165, 166), while verbal reports during mental imagery might additionally distract patients in their mental operations.

Whereas neurofeedback could potentially constitute an objective measure of mental imagery success in the future, variations in applied mental imagery strategies strongly contribute to the heterogeneity of neurofeedback approaches at the moment. In addition to problems that are unspecific to neurofeedback as issues of statistical practice and transparency, particularly, differences in study design and analysis methods have contributed to a pronounced variability between different neurofeedback approaches with fMRI as well as EEG, making it difficult to draw robust clinical or mechanistic conclusions from existing research (see the study by Ros et al. (167) for a contemporary approach to target these issues).

Importantly, a consensus on which criteria should be applied to evaluate success in neurofeedback studies is still lacking. A recent meta-analysis has here made a notable advancement for fMRI neurofeedback by providing an overview on applied success criteria (11) but this information needs to be further utilised to create meta-analytic evidence. Standardising the applied success criteria will thereby enhance the possibility to compare different mental imagery strategies as well as different neurofeedback approaches.

Additionally, heterogeneity in the choice of neural markers used to construct the neurofeedback protocol constitutes a challenge. Most fMRI neurofeedback studies rely on average signals form single regions of interest (e.g., deCharms et al. (119); Haller et al. (168); Zotev et al. (169); Berman et al. (170); Garrison et al. (171); Greer et al. (150)), but several studies have used feedback from activation of whole networks (172, 173), or indices of connectivity between regions (174–177). So far, it is not known if and under which circumstances patients would benefit from training with one marker of neural activity compared to another. Consequently, it is not known whether training a clinically relevant subprocess with a limited number of associated brain regions should be preferred over training distributed pattern of brain activation that better represent the holistic character of an emotional experiences.

Furthermore, the technical set-up of a neurofeedback system additionally creates variance between studies as it determines which markers of brain activation can be extracted: fMRI is characterised by a poor temporal resolution and can therefore not differentiate between neural events that occur close in time. Furthermore, fMRI neurofeedback represents activation that occurred about six seconds before presentation of the feedback. While this delay itself is not necessarily disadvantageous for gaining control over regional brain activation compared to fast changing EEG neurofeedback (178), it constrains fMRI with regard to the information that can be used to create neurofeedback. It remains to be evaluated whether EEG neurofeedback would be preferable for certain psychiatric conditions in which the temporal information contained in the neurofeedback signal has a crucial impact on the treatment outcome.

### Further Technical Advances

While this review focussed on combinations between neurofeedback and mental imagery, another major class of neuromodulation techniques that bear potential for modulating brain systems implicated in mental imagery are brain stimulation approaches. These are technical set-ups that stimulate relevant brain areas using electromagnetic currents. Such neurostimulation techniques can generally be divided into invasive approaches, i.e., approaches that require surgery, and non-invasive approaches, that do not. Considering the risks implicated in neurosurgical procedures such as inflammation (179) and the need for restrictive definition of clinical indications, non-invasive neurostimulation techniques are generally more common. However, non-invasive brain stimulation approaches cannot stimulate deep brain structures reliably, with the possible exception of recently developed techniques for focused transcranial magnetic stimulation (180–182).

Due to the problem that deep brain structures can only be reached with invasive brain stimulation techniques, research is considerably sparse with regard to stimulations of subcortical areas during mental imagery but effects of deep brain stimulations on emotional processes have been demonstrated (183, 184). A major role for neurostimulation would therefor entail modulations of cortical areas that can be penetrated with non-invasive brain stimulation techniques. Several studies have shown that interactions between cortical control regions and lower level visual (185, 186) as well as affective areas (187–190) can be evoked through neuromodulation approaches, and stimulation of the dlPFC has been shown to improve the efficacy of emotion regulation (191). While the mechanistic effects of prefrontal and parietal cortex stimulation remain poorly understood (192), stimulation of lateral prefrontal areas has successfully been implemented as add-on treatment for depression (193, 194), furthermore supporting the strong potential of neuromodulation techniques to contribute to treatments of major psychopathologies. Considering that particularly transcranial-magnetic stimulation (TMS) has been found to be effective for depression, its combination with cognitive behavioural therapy may be promising in this respect (195).

Another direction towards improved interactions between mental imagery and neuromodulation techniques can be expected from approaches that combine neurofeedback with neurostimulation. In such setups a neural target state is achieved by recording brain activation and using information about the current brain state to guide stimulation of brain in order to achieve a certain target state (196–198). In this way, brain stimulation could be used to support the effects of mental imagery during neurofeedback. For example, brain stimulation of limbic areas could help to increase the affective effects of mental images. Moreover, applying brain stimulation and neurofeedback-guided mental imagery successively bears potential. For example, relevant neural systems could first be strengthened with neurostimulation, which does not require active participation of patients. After dysfunctional neural systems have been restabilized, subsequent mental imagery/neurofeedback training could be used to train emotional self-regulation skills and thereby build resilience.

## Discussion

### Summary

The empirical evidence on mental imagery-based psychotherapeutic interventions supports a role for therapeutic approaches targeting/employing mental imagery in affective and anxiety disorders. As mental imagery can induce emotional experiences by triggering perceptual and memory system components of affective states, while at the same time providing the possibility for goal directed self-regulation, mental imagery can modulate a range of clinically relevant mechanisms. Moreover, a broad range of neuroscientific studies already provides an extensive knowledge based on how the implicated psychological processes are manifested in the brain, opening the door for neuromodulation treatments that facilitate the effects of mental imagery.

From the available neuromodulation approaches, particularly, neurofeedback has already been combined with mental imagery extensively. With the possibility to guide the mental operations to evoke maximal impact on clinically relevant brain systems and the possibility to reinforce associated brain states, neurofeedback provides interesting synergistic opportunities with imagery-based psychological interventions. The beneficial interplay between neuromodulation, cognitive self-regulation, and mental imagery, although this field is still in the early phases of clinical evidence-gathering.

Because mental imagery is one of the most common mental strategies applied during neurofeedback, a much larger amount of empirical research exists for combinations of mental imagery with neurofeedback compared to other neuromodulation techniques. Although neurofeedback protocols often incorporate mental strategies which bear resemblance to psychotherapeutic approaches (such as exposure or positive mental imagery), these parallels have not been exploited so far in a systematic fashion or through combined treatment manuals. Such exploitation remains an attractive task for the collaboration of clinical psychologists, psychiatrists, and neuroscientists.

### Conclusion

In conclusion, mental imagery is a core process of many psychological interventions, particularly in affective and anxiety disorders. It has also been a core topic of investigation of cognitive neuroscience, and the neural and psychological mechanisms of mental imagery and its impact on emotional experience have been extensively studied. Based on this knowledge of processes, we can now exploit synergies between imagery and neuroscientific techniques, in order to use the latter to boost the former, or use the former to facilitate the latter. This combination is inherent in many neurofeedback protocols, particularly those employing fMRI signals, but has also been studied with non-invasive brain stimulation. The combination of mental imagery with neuromodulation is conceptually attractive because of the synergistic potential and the potential to achieve more sustainable effects for neurostimulation interventions through transfer to psychological processes.

Yet, the potential treatment combinations are manifold, and the same is true for the neuropsychological subprocesses that could be targeted, so that an extensive body of research is still needed in order to determine the most effective treatment combinations and parameters for this type of neuropsychotherapy.

## Author Contributions

Both authors designed, drafted and reviewed this review article.

## Funding

The authors were supported by the BRAINTRAIN grant, a Collaborative Project supported by the European Commission, under the Health Cooperation Work Programme of the 7th Framework Programme, under the Grant Agreement no. 602186 (www.braintrainproject.eu).

## Conflict of Interest

The authors declare that the research was conducted in the absence of any commercial or financial relationships that could be construed as a potential conflict of interest.
